# A comprehensive bioinformatics analysis of pathways and biomarkers shared between type 2 diabetes mellitus and chronic obstructive pulmonary disease

**DOI:** 10.3389/fimmu.2025.1536551

**Published:** 2025-07-25

**Authors:** Tingting Hu, Xiaomei Duan, Jiale Gao, Zheng Li, Dan Xu, Jing Jing, Fengsen Li, Jianbing Ding, Li Ma, Min Jiang, Jing Wang

**Affiliations:** ^1^ Clinical Laboratory Center, Traditional Chinese Medicine Hospital Affiliated to Xinjiang Medical University, Urumqi, China; ^2^ Xinjiang Laboratory of Respiratory Disease Research, Traditional Chinese Medicine Hospital Affiliated to Xinjiang Medical University, Urumqi, China; ^3^ Department of Immunology, College of Basic Medicine, Xinjiang Medical University, Urumqi, China; ^4^ Department of Endocrinology, Traditional Chinese Medicine Hospital Affiliated to Xinjiang Medical University, Urumqi, China

**Keywords:** type 2 diabetes mellitus, Chronic Obstructive Pulmonary Disease, weighted gene co-expression network analysis, machine learning, single-cell sequencing, SUMF2

## Abstract

**Background:**

T2DM and COPD are prevalent and high-burden diseases which are closely related, with poor patient outcomes. In this study, we aimed to identify common diagnostic markers for T2DM and COPD and their therapeutic potential.

**Methods:**

Microarray data from the GEO database were analyzed to identify DEGs, whereas WGCNA, co-differential gene analyses were employed to identify co-expression modules and DEGs functions. Diagnostic markers were determined through machine learning and validated with human blood PBMC and single-cell sequencing.

**Results:**

A total of 738 and 1391 DEGs were identified for T2DM and COPD, respectively. Among these, 25 key genes and 75 co-differential genes were recognized, predominantly enriched in immune-related pathways, particularly those involving T-cell signaling. Eight diagnostic markers were identified through machine learning approaches. Subsequent validation using human PBMC from three groups (Ctrl, COPD, and T2DM, n=15 each) confirmed PES1 (AUC 0.676 and 0.615), CANX (AUC 0.668 and 0.642), SUMF2 (AUC 0.684 and 0.679), and DCXR (0.625 and 0.606) as shared diagnostic markers. Analysis of single-cell sequencing data from blood and bone marrow and RT-qPCR results from healthy individuals and patients with T2DM combined with COPD showed that only SUMF2 showed a statistically significant difference in expression levels in comorbid patients and was strongly associated with T-cell subpopulations.

**Conclusion:**

The T-cell pathway may be involved in the pathogenesis of T2DM and COPD, and SUMF2 may be a potential diagnostic marker, and its high expression in T-cell subsets suggests a possible role in the immunomodulatory mechanisms underlying the two diseases.

## 1 Introduction

Presently, the global prevalence of diabetes is ~1/11 adults [of which 90% have Type 2 Diabetes Mellitus (T2DM)], with Asia as the epicenter of the global T2DM epidemic (1). Various factors have been linked to the etiology of T2DM. Among them is chronic inflammation, which could lead to insufficient insulin secretion in the body or the body’s inability to efficiently utilize insulin, resulting in persistently elevated blood glucose levels (2, 3). With increased socioeconomic developments, the T2DM incidence rate has been on the rise each year. This phenomenon implies increased incidences of long-term elevation in blood glucose levels, which could severely damage various organs directly and indirectly cause multiple blood vessel damage-related complications, seriously compromising patients’ quality of life (4). On the other hand, Chronic Obstructive Pulmonary Disease (COPD), a prevalent chronic respiratory disease, is characterized by persistent respiratory symptoms and airflow limitations resulting from airway and/or alveolar abnormalities (5). According to research, inflammation is crucially involved in the pathogenesis of COPD; hence, the disease could also be defined as a complex chronic airway inflammatory complication resulting from reactions involving multiple inflammatory cells and chemotactic factors (6). A large body of literature has established that COPD is a systemic disease with multiple co-morbidities, and T2DM is a common co-morbidity. It has been found that patients with COPD have an 8.2% increased risk of developing T2DM compared to the general population respectively (7). Meanwhile, patients with comorbid COPD are usually accompanied by longer hospitalization and poorer prognosis compared to patients with T2DM alone, and the risk of poor prognosis increases with decreasing lung function (8). These phenomena suggest that the two may share a pathophysiological basis that transcends traditional categorization, but existing studies have remained at the level of phenotypic associations on the mechanisms of interaction between the two and lacked in-depth exploration of potential common genetic features.

From a pathomechanistic perspective, T2DM and COPD show a surprising convergence in multiple biological pathways, and chronic inflammation is a central feature of both, thus focusing on resolving the bridging role of specific diagnostic markers in the -immune-inflammatory axis is of clinical importance for early therapeutic intervention in patients with T2DM combined with COPD. In this study, we reviewed published gene expression data from the GEO database and used a systems biology approach to explore the potential role of shared gene pathways and diagnostic markers in immune regulation between T2DM and COPD. Utilizing these data to reveal the molecular nature of this “cross-systems dialogue” will not only help to understand the overall regulatory network of chronic diseases, but may also provide new perspectives for the development of cross-disease therapeutic strategies.

## 2 Materials and methods

### 2.1 Data collection and pre-processing

Herein, the GSE184050, GSE21321, GSE56766, and GSE42057 GEO datasets were used. Sequencing was performed on Peripheral Blood Mononuclear Cells (PBMCs). The GSE184050 dataset comprised 116 samples (50 and 66 peripheral blood samples from T2DM patients and healthy controls, respectively). Furthermore, the GSE21321 dataset comprised 17 samples (9 and 8 peripheral blood samples from T2DM patients and healthy controls, respectively). On the other hand, the GSE56766 dataset contained 204 samples (137 and 67 peripheral blood samples from COPD patients and healthy controls, respectively). Finally, the GSE42057 dataset comprised 136 samples (94 and 42 peripheral blood samples from COPD patients and healthy controls, respectively). We used ComBat for batch effect correction, adata = adata[adata.obs.pct_counts_mt < 10],: mitochondrial genes expressed less than 10% adata = adata[adata.obs.n_genes_by_counts < 4000],: gene counts expressed less than 4000 Normalized using normalize_total, log1p for logarithmic, highly_variable_genes to screen for TOP2000 highly variable genes, used harmony to remove batch effects.

### 2.2 Differential genetic screening

We screened the T2DM datasets GSE184050 and GSE21321 and the COPD datasets GSE42057 and GSE56766 for their respective co-DEGs using the Limma R software package. The cut-off criterion was p. adj. value < 0.05.

### 2.3 Functional enrichment analysis of core genes

The core gene set associated with T2DM and COPD comprised key genes derived from the DEGs. Herein, we aimed to determine the comorbidity mechanism between T2DM and COPD and elucidate the potential molecular biological processes underlying the core genes of the diseases. Key biological mechanisms and functions were identified using Kyoto Encyclopedia of Genes and Genomes (KEGG) pathway enrichment analysis, which was performed using the clusterProfiler package in R, wherein results with p < 0.05, q < 0.05, and a higher Gene Ratio were considered more significant.

### 2.4 Construction and module analysis using weighted gene co-expression Network Analysis

The GSE184050 and GSE56766 datasets were subjected to WCGNA using the R.4.0.3 package. Co-expression networks with corresponding clinical characteristics for the DEGs of T2DM and COPD were constructed using the WGCNA package in R. Before the analysis, hierarchical cluster examination was performed using the Hclust function in R language to exclude outlier samples. Subsequently, the “pickSoftThreshold” function in the WGCNA software package was used to select the appropriate soft power b (ranging from 1 to 20) per the scale-free network standard for automatic network construction. The results were clustered using Topological Overlap Matrix (TOM) analysis, which includes module assignments labeled by color and Module Eigengene (ME). Furthermore, Pearson’s correlation analysis was employed to determine the correlation between ME and clinical features. Finally, the modules most relevant to T2DM and COPD were screened (p-value < 0.05).

### 2.5 Gene set variation analysis

We scored the cellular senescence pathway using the R package “GSVA 1.36.2”. The GSVA scoring was done in a non-parametric way using a K-sample randomized wandering statistic and sample-specific as well as genome-specific negative values.

### 2.6 Functional enrichment analysis

Herein, Gene Ontology (GO) enrichment analysis, a commonly used bioinformatics method for searching and analyzing comprehensive large-scale genetic data, was employed.

### 2.7 Identification of diagnostic markers using machine learning methods

First, DEGs shared between T2DM and COPD were identified. Following that, ML methods were used to analyze the GSE56766 and GSE184050 datasets to predict core pathogenic genes in T2DM combined with COPD. Three ML methods, including LASSO regression, the Random Forest (RF) method, and the Support Vector Machine (SVM) method, were employed for feature selection and model training. The SVM algorithm was implemented using the e1071 package (9). To calculate the error in the training queue, 10-fold cross-validation was used to determine the algorithm’s accuracy. First, diagnostic markers in the T2DM and COPD datasets were obtained, with their overlapping section between the two diseases representing the shared diagnostic markers. Additionally, the diagnostic effectiveness of the core markers in the modeling and validation sets of both T2DM and COPD was evaluated using Area Under the Receiver Operating Characteristic (AUROC) curve values.

### 2.8 PBMC extraction

Using EDTA anticoagulation tube, 2 mL blood samples were collected from T2DM patients, COPD patients, COPD patients with T2DM, and healthy controls. Subsequently, the human peripheral blood and Lymphocyte Isolation Solution were mixed homogeneously at a ratio of 1:5 (2 mL:10 mL), incubated on ice for 15 min, vortexed and mixed twice, and centrifuged at 2100 rpm for 10 min at 4°C, after which the supernatant was removed. Following that, 4 mL Lymphocyte Isolation Solution was added again to the leukocyte precipitate, centrifuged at 2100 rpm for 10 min at 4°C, and then the supernatant was removed and used to obtain PBMCs. The Ethics Committee of the Affiliated Hospital of Traditional Chinese Medicine of Xinjiang Medical University approved the aforementioned procedures and all patients signed an informed consent form. **Table 1** presents detailed information on the demographic characteristics of the patients.

**Table 1:** Participant demographic characteristics.

Participant	Ctrl	T2DM	COPD	COPD+T2DM
n	25	15	15	10
Age (yr)	53.1 ± 3.3	54.4 ± 3.3	54.1 ± 3.6	58.5 ± 6.3
Gender (M/F)	16/9	7/8	14/1	3/7
FPG (mmol/L)	4.8 ± 0.6	10.8 ± 2.6	4.7 ± 0.5	10.1 ± 1.7
FEV1/FVC (%)	82.4 ± 3.6	83.8 ± 4.5	60.0 ± 5.3	59.8 ± 5.7

### 2.9 RNA extraction and quantitative polymerase chain reaction analysis

First, total RNA was extracted using the RNAprep Pure Hi-Blood Kit (TIANGEN, CHINA). Using the PrimeScriptTM RT Reagent Kit (Takara), we then performed Real-time quantitative PCR (RTqPCR) on an ABI 7500 fast real-time PCR system (Thermo Fisher Scientific, USA) to determine the target RNA expression levels. The PCR conditions of the PrimeScriptTM RT Reagent Kit were as follows: 95°C for 3 min, followed by 40 cycles of 60°C for 30s and 72°C for 10s. The relative quantity of the target gene was determined using the 2^−△△Ct^ method and normalized with GAPDH. **Table 2** shows the primer sequences used.

**Table 2:** Primers for the reverse transcription PCR analysis of genes.

Gene	Primer	Primer Sequence (5’-3’)
CANX (ms)	Forward primer Reverse primer	TGGTTATCCTCTTCTGCTGTTCTGG TCTTCTCCTTCCTCCTCCTCATCTC
DCXR (ms)	Forward primer Reverse primer	CCGTGGACCTGCTGGTGAAC ACAATCTGCGACACCTGGATGAC
GCDH (ms)	Forward primer Reverse primer	CCAGAGCCCACTACAACTCATCC ACACCGAGCCCACACTACAAAC
NOP2 (ms)	Forward primer Reverse primer	GCGTTGCTGCCCATTGAAAGAG CTCCTCGTCCTCGGTCTCCTC
PIP4K2B (ms)	Forward primer Reverse primer	CAGAGGACGAGGAGTGTGAATG AGGACCAAAGAACCGAGGAAAGC
SRM (ms)	Forward primer Reverse primer	ACATCCTCGTCTTCCGCAGTAAG GGTTGGCGATCATCTCCTGGTAG
SUMF2 (ms)	Forward primer Reverse primer	AGGTCCTGGCTCTGGCATCC CGGCAAACTCCCACTCTTCCTC
ACTIN (ms)	Forward primer Reverse primer	CATGTACGYYGCTATCCAGGC CTCCTTAATGTCACGCACGAT
CANX (h)	Forward primer Reverse primer	AAGAGGCCACAAAACCCGAA AGGAGCCTCCCATTCTCCAT
SUMF2 (h)	Forward primer Reverse primer	TCCCCAGTGAATGCTTTCCC CCATCAGCTGTGTCGATCCA
PES1 (h)	Forward primer Reverse primer	ACGTTCCACCTGAGAAGCTG GTCGTCCTCTTCCTCCTCCT
DCXR (h)	Forward primer Reverse primer	GGACATGCTGACCAAGGTGA TGGACGTCATCACCACTGTG
ACTIN (h)	Forward primer Reverse primer	GAGAAGGCTGGGGCTCATTTGC TGCTGATGATCTTGAGGCTGTTGTC

ms, mouse; h, human.

### 2.10 Immune analysis algorithm

Based on the expression levels of immune cell-related genes, the CIBERSORT algorithm was used to calculate the proportion of different immune cell types. The results of 22 infiltrating immune cells were then integrated, and the component matrix of immune cells was generated for analysis. Based on the immune infiltration analysis results of the above-mentioned common markers, the correlation between the core markers and the expression of immune infiltrating cells was analyzed using the nonparametric correlations (Spearman’s) method.

### 2.11 Data processing and analysis for the single-cell transcriptome

First, three datasets were downloaded from the GEO database (GSE216886 and GSE212726 for T2DM and GSE205078 for COPD). The GSE216886 dataset comprised of two samples, one each from normal mice whole blood and T2DM mice whole blood. Similarly, the GSE212726 dataset comprised of two samples, one each from normal mice bone marrow cells and T2DM mice bone marrow cells. On the other hand, the GSE205078 dataset comprised six samples including one whole blood sample and one bone marrow cell sample from each of the three mice groups (Healthy controls, T2DM, and COPD). In single-cell transcriptome data processing, cells were first normalized and then scaled and clustered using the Python library “scanpy” to obtain 12 major cell types.

Single cells were extracted based on a threshold of nFeature_RNA<4000 and mitochondrial genes < 10%. The filtered gene barcode matrix was normalized using the “LogNormalize” method and the “NormalizeData” function. The first 2000 highly variable genes were identified using the “vst” method and the FindVariableFeatures function, which was previously centered and scaled using “ScaleData”. Following that, Principal Component Analysis (PCA) was performed based on the 2000 highly variable genes. Dimensionality reduction was then performed using the Harmony package to remove batch effects. Subsequently, clusters with dimensionality reduction were displayed on a 2D map generated using Scanpy’s “FindNeighbors”, “FindClusters” and “umap” functions. Differences in gene expression were estimated using the Wilcoxon test.

### 2.12 Statistical analysis

All statistical analyses were performed using R software (version 3.6.2). Gene expression levels across clinical samples were compared using the Student t-test. Results with p < 0.05 were considered statistically significant.

## 3 Results

### 3.1 Screening of T2DM DEGs

**Figure 1:**
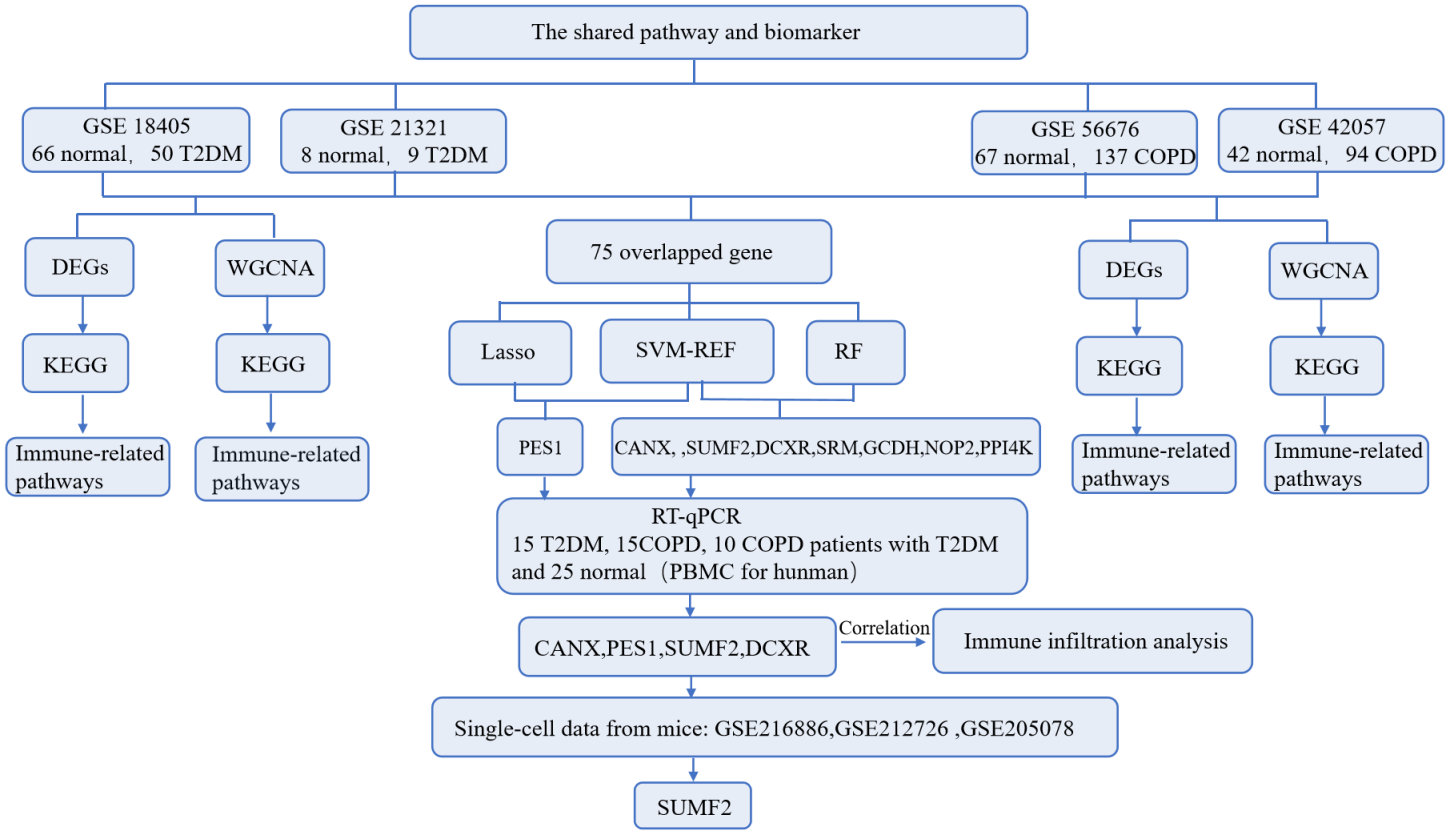
The complete study workflow.

**Figure 2:**
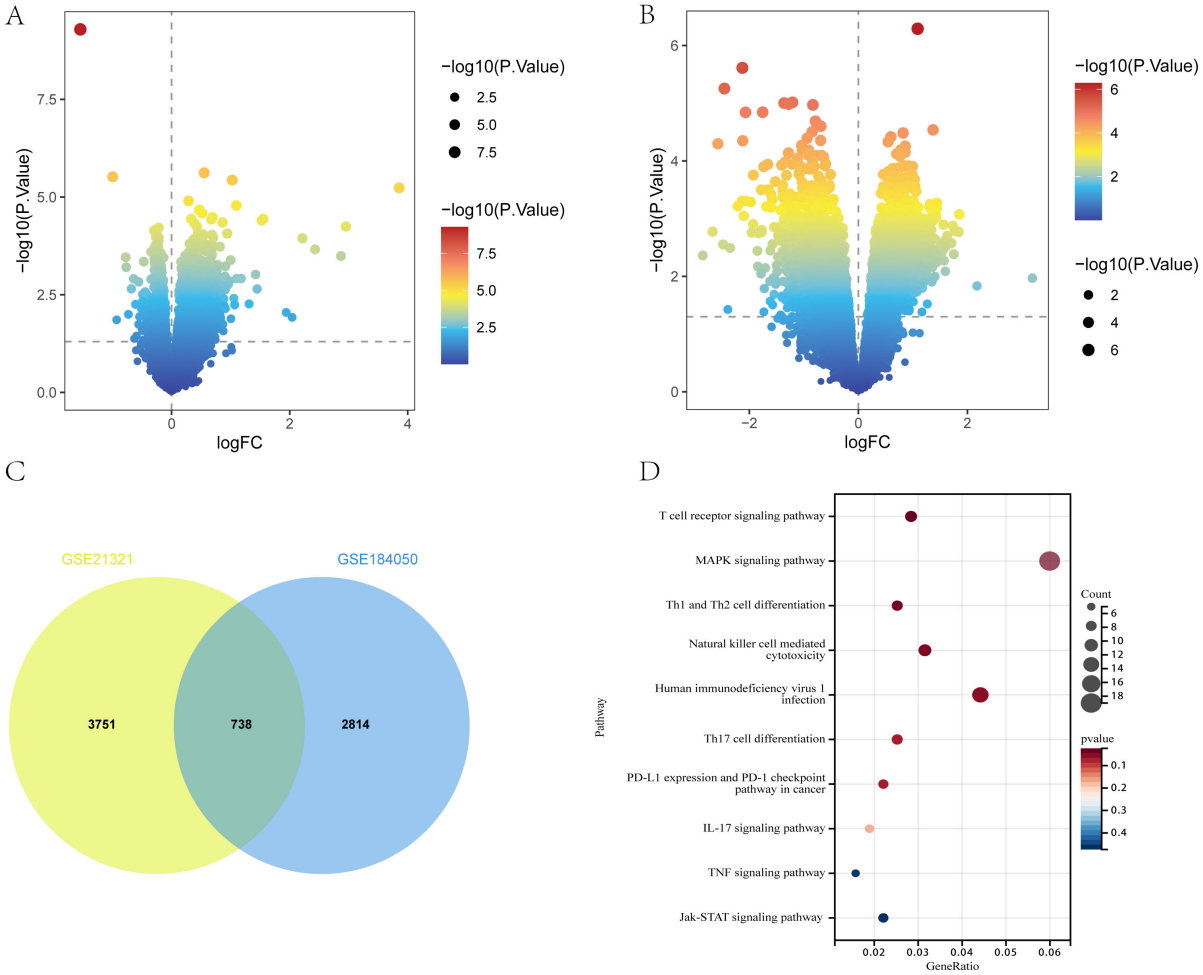
DEGs of T2DM. **(A, B)** Volcano plot of DEGs in GSE184050 and GSE21321 (*p* < 0.05). **(C)** Venn diagram of shared DEGs in GSE184050 and GSE21321. **(D)** Bubble chart of KEGG enrichment analysis of shared DEGs.

The flow of this study is shown in **Figure 1**. A total of 3552 and 4489 DEGs were identified between T2DM patients and healthy controls in the GSE184050 and GSE21321 datasets, respectively. Among the 3552 DEGs in the GSE184050 dataset, 2526 and 1026 were upregulated and downregulated, respectively (**Figure 2A**). Furthermore, of the 4489 DEGs in the GSE21321 dataset, 2592 and 1897 were upregulated and downregulated, respectively (**Figure 2B**). The intersection of the two datasets comprised 738 common DEGs (**Figure 2C**), which were subjected to KEGG enrichment analysis. According to the results, the DEGs were mostly enriched in immune-related pathways, especially those associated with Th1, Th2, and Th17 cell differentiation (**Figure 2D**).

### 3.2 Screening of COPD DEGs

**Figure 3:**
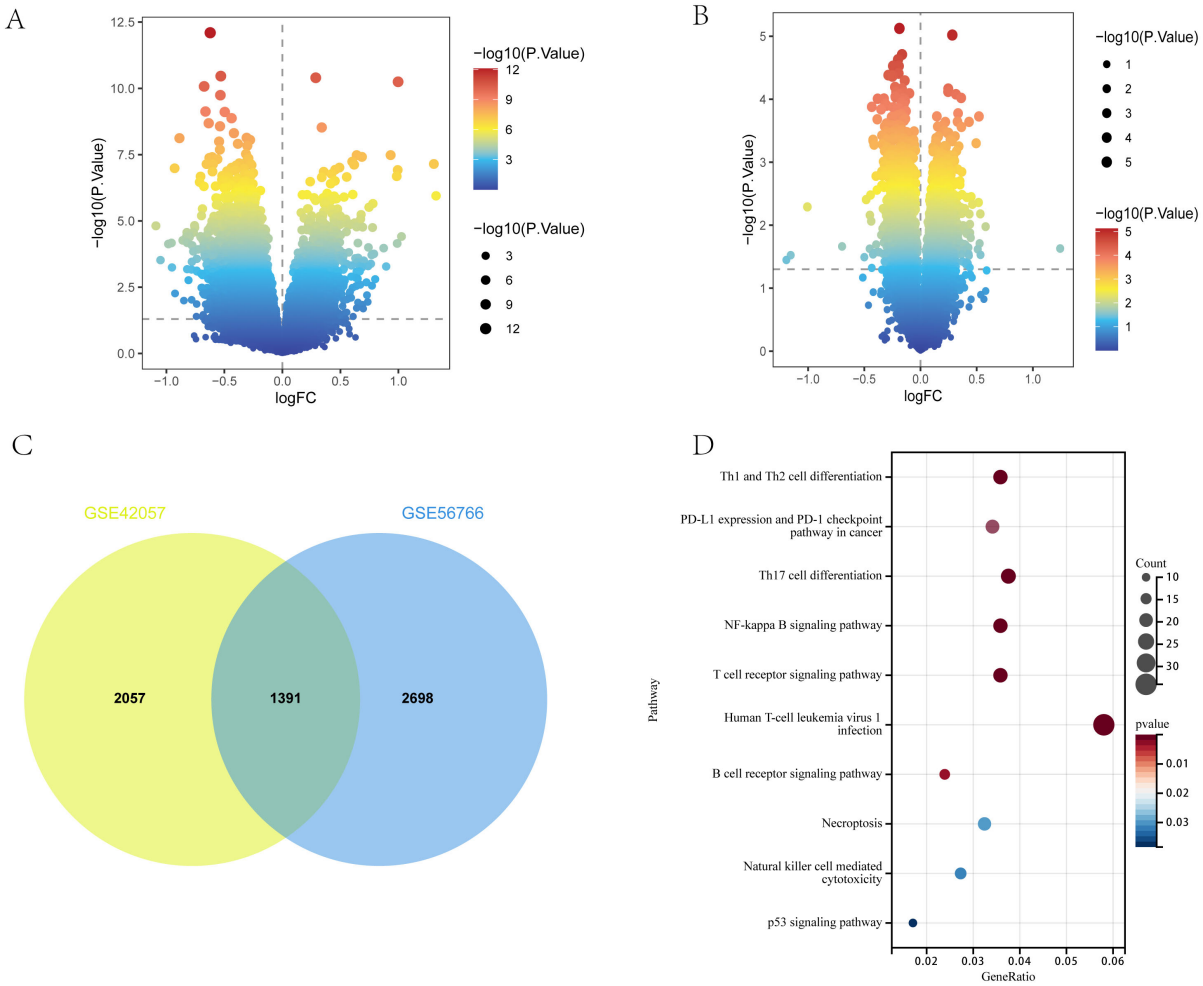
DEGs of COPD. **(A, B)** Volcano plots of DEGs in GSE56676 and GSE42057 (*p* < 0.05). **(C)** Venn diagram of shared DEGs in GSE56676 and GSE42057. **(D)** Bubble chart of KEGG enrichment analysis results of shared DEGs.

Between COPD patients and healthy controls, 4149 and 3448 DEGs were identified in the GSE56676 and GSE42057 datasets, respectively. Of the 4149 DEGs in the GSE56676 dataset, 1267 and 2822 were upregulated and downregulated, respectively (**Figure 3A**). Furthermore, of the 3448 DEGs in the GSE42057 dataset, 1348 and 2100 were upregulated and downregulated, respectively (**Figure 3B**). The intersection of the two datasets comprised 1391 shared DEGs (**Figure 3C**), which were subjected to KEGG enrichment analysis. Consistent with the KEGG enrichment analysis results for T2DM DEGs, COPD DEGs were mostly enriched in immune-related pathways, especially those associated with Th1, Th2, and Th17 cell differentiation (**Figure 3D**).

### 3.3 WGCNA establishment and module analysis

**Figure 4:**
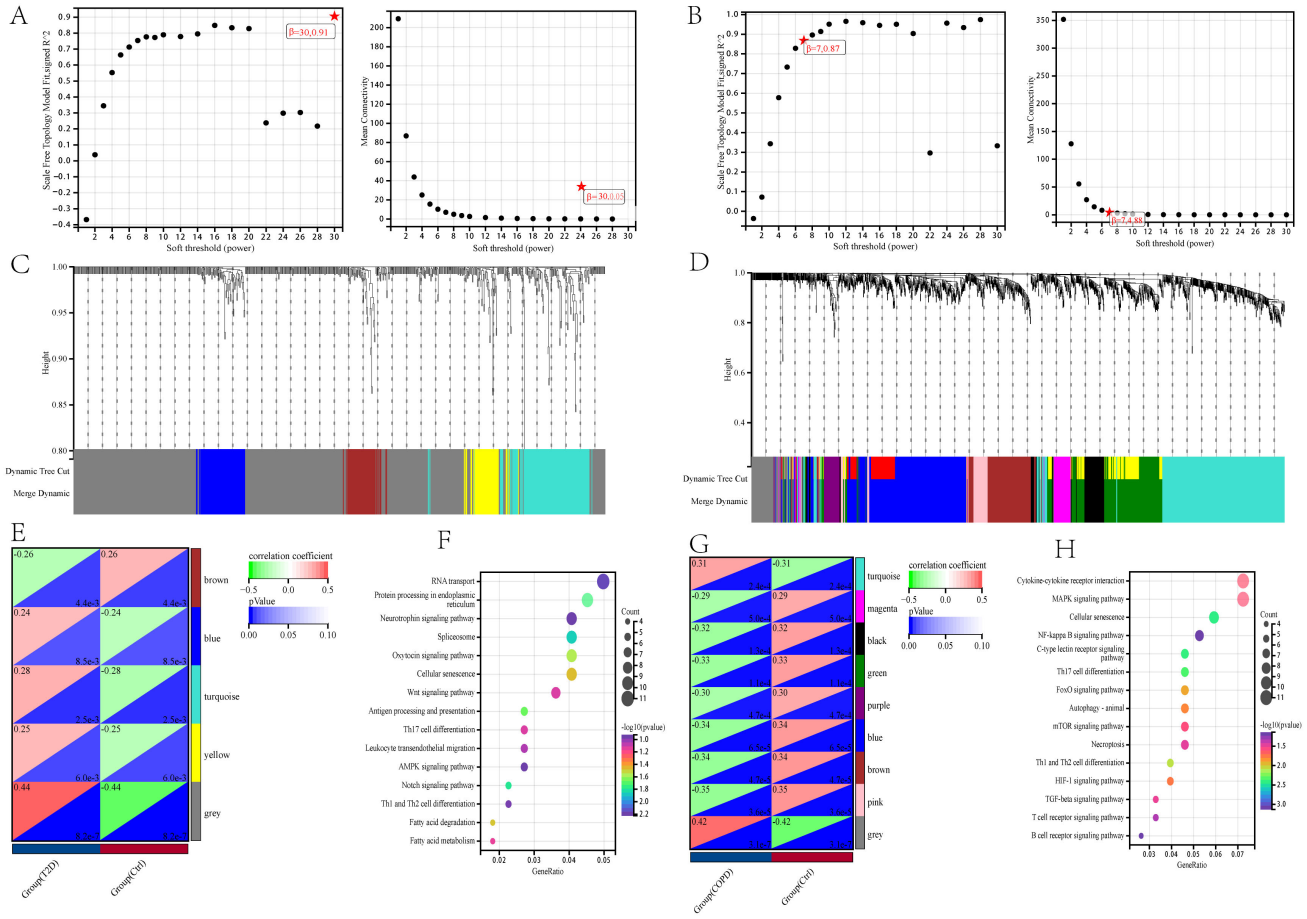
Construction of weighted co-expression network related datasets and identification of related key modules in T2DM (GSE18405) and COPD (GSE56676). **(A, B)** Network topology analysis for various soft thresholds (β). The left figure shows the scale-free fitting index (scale-independent, y-axis) as a function of soft-threshold power (x-axis); the right figure shows the average connectivity (degree, y-axis) as a function of soft-threshold power (x-axis). **(C, D)** Gene dendrogram obtained by average chained hierarchical clustering. The colored rows below the dendrogram shows the module assignments determined by dynamic tree-cutting method. **(E, G)** The module-trait relationships: each row in the heatmap correspond to a ME and each column to a clinical trait. Each cell contains the corresponding correlation and p-value. **(F, H)** KEGG enrichment analysis of all genes in the co-expressed gene modules of grey and brown for T2DM and grey and pink for COPD.

The DEG clusters shared between T2DM and COPD were identified using WGCNA and correlations between the combined modules and disease characteristics were determined. First, to ensure biologically meaningful scale-free networks, based on an R2 scale independence > 0.85 and an average connectivity converging to 0, 30 and 7 were selected as the optimal soft threshold power β for the T2DM and COPD datasets, respectively (**Figures 4A–D**). Second, after merging similar gene modules, five and nine modules were identified in the T2DM and COPD models, respectively. The grey module had the strongest positive correlation with T2DM occurrence (r=0.44), while the brown module had the strongest negative correlation with T2DM occurrence (r=-0.26) (**Figure 4E**). Additionally, in the COPD modeling set, the grey module exhibited the strongest positive correlation with COPD (r=0.42), while the pink module had the strongest negative correlation with COPD (r=-0.35) (**Figure 4G**). These modules could be regarded as co-expressed gene modules closely related to the combined complication of T2DM and COPD.

All the genes in the grey and brown co-expressed gene modules in T2DM and the grey and pink co-expressed gene modules in COPD were subjected to KEGG enrichment analysis. The results showed that DEGs, whether related to T2DM or COPD, were mostly more enriched in immune-related pathways, especially those related to Th1, Th2, and Th17 cell differentiation, in addition to focusing on cellular senescence (**Figures 4F, H**), by GSVA analysis we found that cellular senescence scores were lower in both T2DM and COPD, so after that we focused on immune(**Supplementary Figure 2**).

### 3.4 DEGs shared between T2DM and COPD

**Figure 5:**
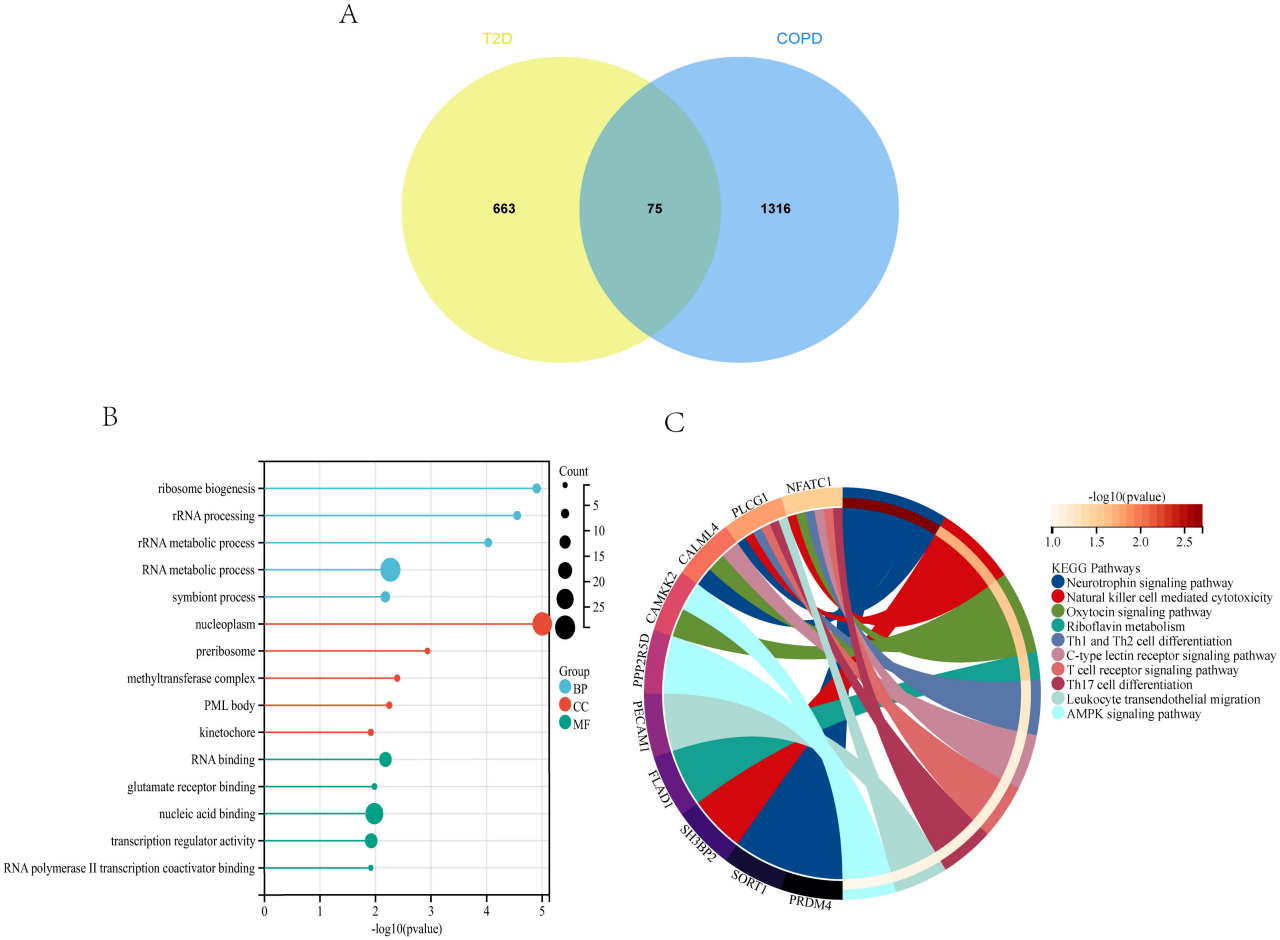
T2DM and COPD DEGs analysis **(A)** Venn diagram of shared DEGs of T2DM and COPD. **(B, C)** GO and KEGG enrichment analysis of shared DEGs of T2DM and COPD.

Analysis of the genes shared between T2DM and COPD yielded 75 DEGs (**Figure 5A**), which were subjected to GO enrichment analysis using the clusterProfiler package in R (**Figure 5B**). The analysis yielded 879 GO terms, including 682 Biological Processes (BPs), 101 Cellular Components (CCs), and 96 Molecular Functions (MFs). The core genes were mainly enriched in the RNA metabolic process and nucleoplasm for BPs and CCs, respectively, and in nucleic acid binding for MFs.

The 75 DEGs were also subjected to KEGG pathway analysis, revealing that the core genes were mainly enriched in the Neurotrophin signaling pathway, Natural Killer (NK) cell-mediated cytotoxicity, Th1 and Th2 cell differentiation, and other immune-related pathways (**Figure 5C**). Overall, T2DM and COPD shared many molecular mechanisms, of which the majority were closely related to immunity, mainly encompassing Th1, Th2, and Th17 cell differentiation.

### 3.5 Identification of potential shared diagnostic genes

**Figure 6:**
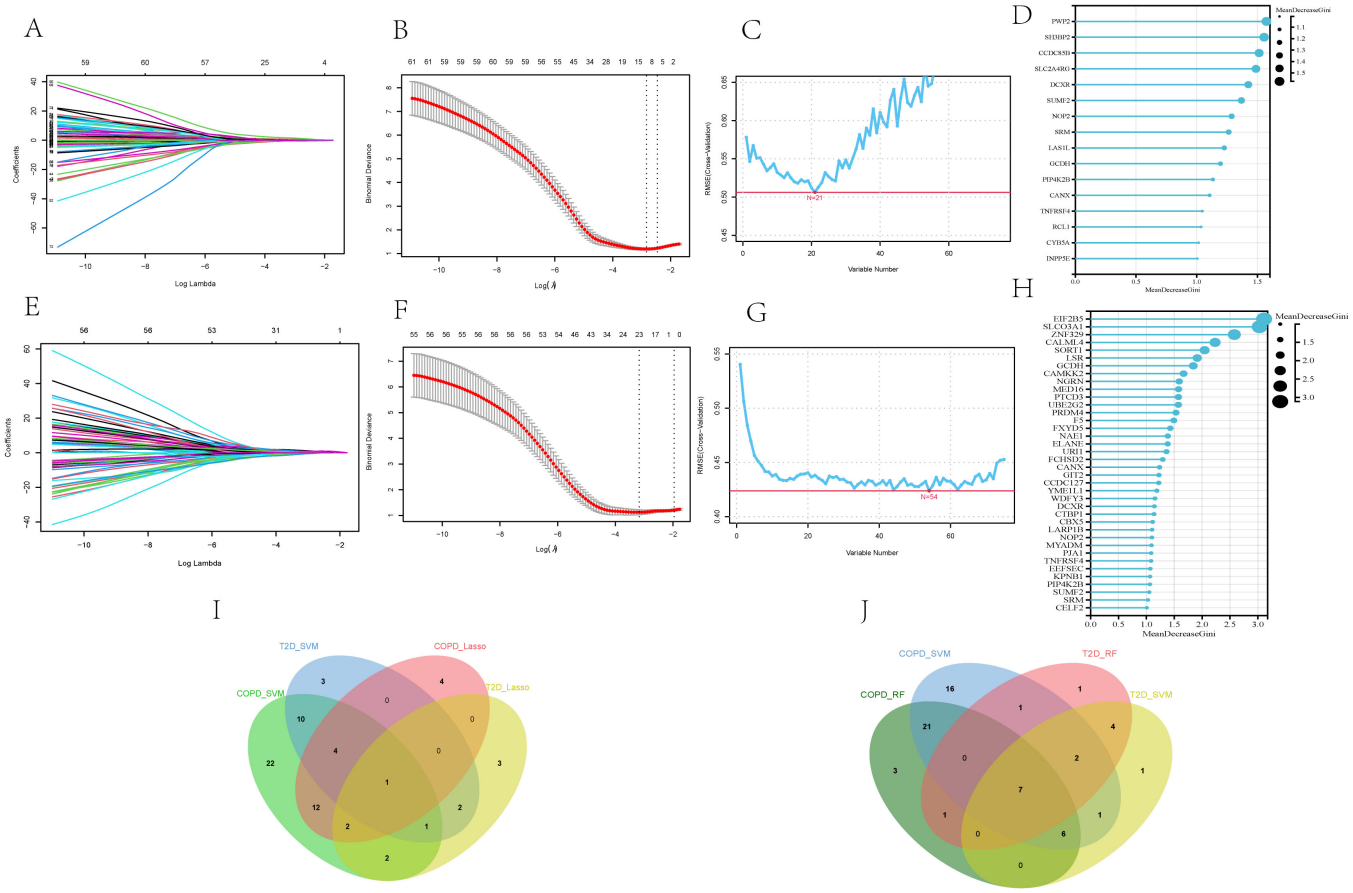
Screening of core genes of T2DM and COPD by machine-learning approaches. **(A, B)** Diagnostic markers in the GSE18405 of T2DM identified by LASSO regression algorithm. **(C, D)** Diagnostic markers in the GSE18405 of T2DM identified by SVM-RFE and RF. **(E, F)** diagnostic markers of the GSE56676 of COPD identified by LASSO regression algorithm. **(G, H)** Diagnostic markers of GSE18405 of COPD identified by SVM-RFE and RF. **(I)** Venn diagram of PES1, the core gene of T2DM and COPD identified by LASSO and SVM-RFE. **(J)** Venn diagram of seven core genes of T2DM and COPD identified by RF and SVM-RFE, including CANX, DCXR, GCDH, NOP2, PIP4K2B, SRM and SUMF2.

The 75 core genes related to both T2DM and COPD were further subjected to LASSO regression, SVM-RFE, and RF analyses to screen for disease-related diagnostic markers. After 10-fold cross-validation, for T2DM diagnosis, LASSO regression, SVM-RFE, and RF identified 11, 21, and 16 core genes, respectively (**Figures 6A–D**). On the other hand, for COPD diagnosis, LASSO regression, SVM-RFE, and RF identified 23, 54, and 38 core genes, respectively (**Figures 6E–H**). The shared core genes for the diagnosis of T2DM and COPD were considered diagnostic markers of the combined complication of T2DM and COPD. The intersection of LASSO regression and SVM-RFE revealed that Pescadillo ribosomal biogenesis factor1(PES1) was a diagnostic marker gene, while the intersection of RF and SVM-RFE comprised seven diagnostic markers, including Calnexin (CANX), dicarbonyl and L-xylulose reductase (DCXR), Glutaryl-CoA Dehydrogenase (GCDH), NOP2 Nucleolar Protein (NOP2), Phosphatidylinositol-5-Phosphate 4-Kinase Type 2 Beta (PIP4K2B), Spermidine Synthase (SRM), and sulfatase-modifying factor (SUMF2) (**Figures 6I, J**).

**Figure 7:**
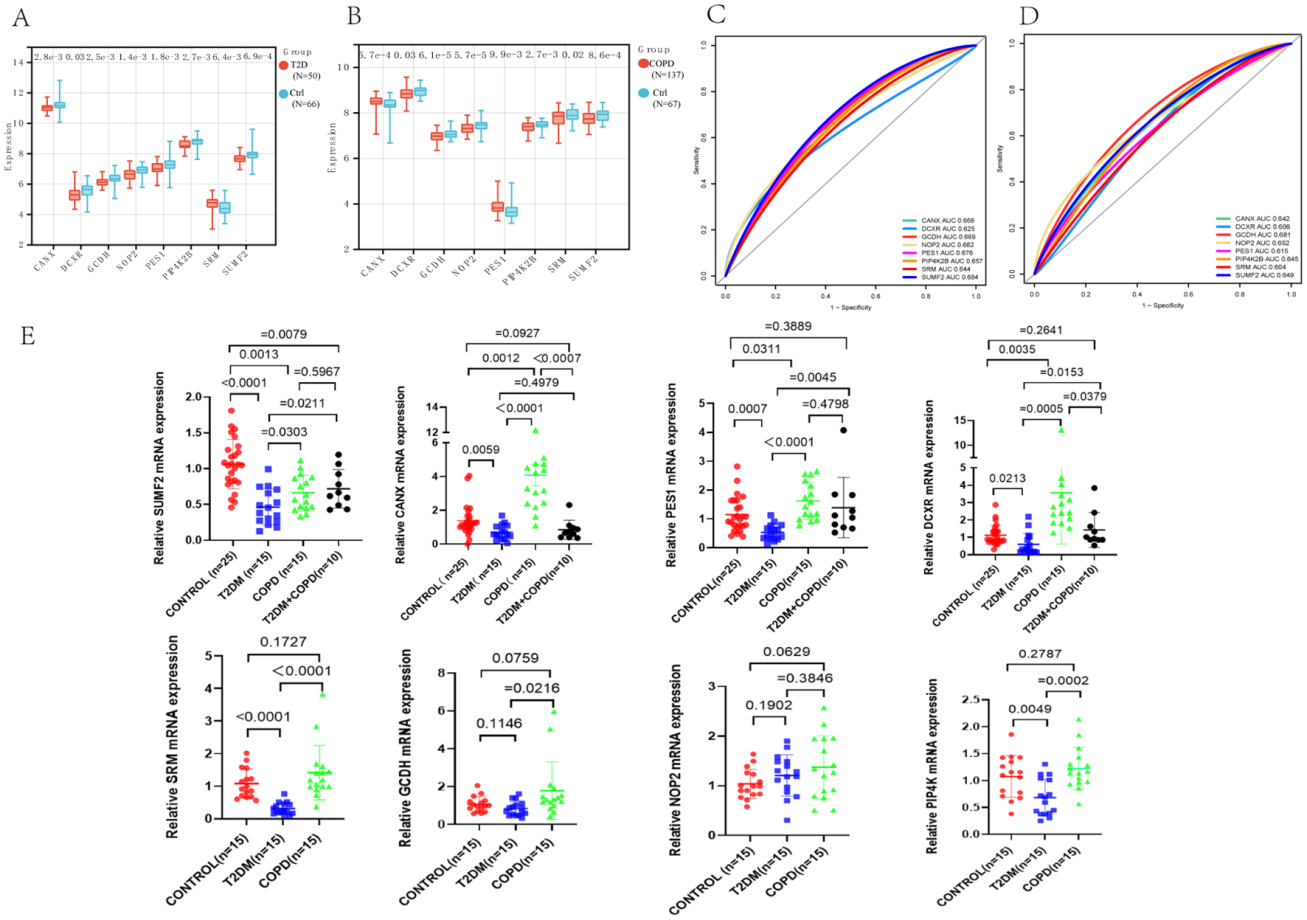
Identification of core genes of T2DM and COPD. **(A, B)** PES1, CANX, DCXR, GCDH, NOP2, PIP4K2B, SRM and SUMF2 showed significant differences in GSE18405 and GSE56676. **(C, D)** ROC curves of eight core genes. **(E)** Real-time fluorescence quantitative PCR analysis of mRNA expression levels of PES1, CANX, DCXR, GCDH, NOP2, PIP4K2B, SRM and SUMF2 in PBMCs from patients and healthy control.

The expression levels of these genes in the two disease datasets were then analyzed (**Figures 7A, B**). According to the results, the T2DM and COPD models exhibited different gene expression levels compared to healthy controls. Specifically, most diagnostic marker genes in the T2DM and COPD models showed lower expression levels compared to healthy controls. Additionally, the ROC curves of the diagnostic indicators were plotted in R Studio to determine their diagnostic values, revealing that the eight diagnostic markers screened exhibited significant diagnostic values in disease classification (**Figures 7C, D**).

Furthermore, fresh whole blood samples were collected from 65 individuals (including 25 healthy individuals, 15 each from T2DM patients and 15 COPD patients, and 10 from T2DM combined with COPD patients). PBMCs were then extracted and analyzed using RT-qPCR to further confirm differential expression of the identified genes in the patient samples. The results showed that the expression trends of PES1, CANX, SUMF2 and DCXR were consistent with the above predictions, especially SUMF2, which was not only reduced in T2DM and COPD patients, but also significantly reduced in patients with comorbidities compared with healthy controls. Meanwhile, we found an interesting phenomenon that the expression levels of all genes, except NOP2, were significantly higher in COPD patients than in T2DM patients (**Figure 7E**).

### 3.6 Immunocyte correlation analysis

**Figure 8:**
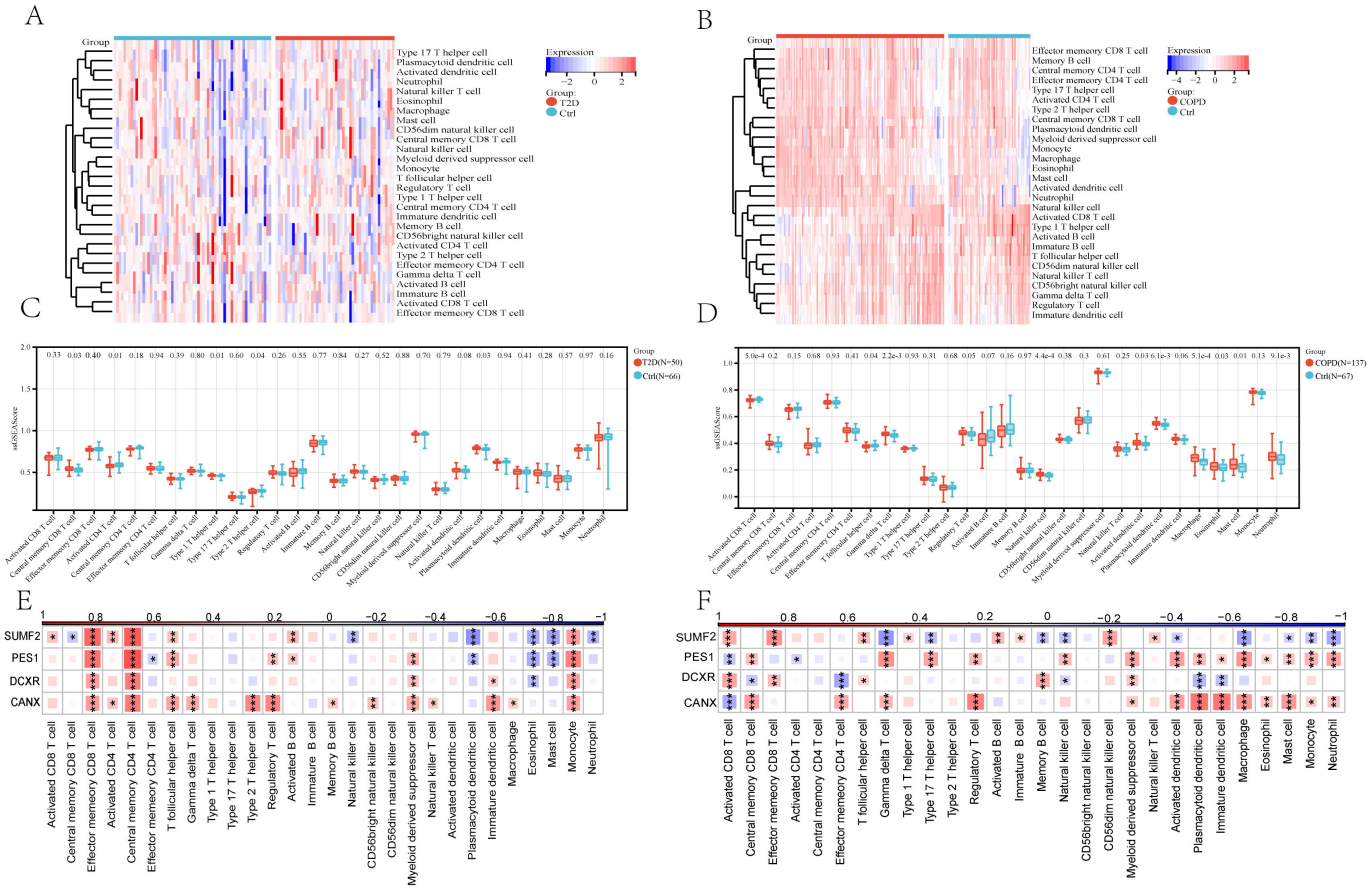
The 28 immunocytes in cases with both T2DM and COPD and correlation analysis of 28 immunocytes and four core genes. **(A, B)** Heatmap of 28 immunocytes expression scores in T2DM and COPD. **(C, D)** Comparison of the scores of 28 immunocytes in Ctrl and T2DM samples and Ctrl and COPD samples. **(E, F)** Spearman correlation analysis of 4 common core genes with 28 immunocytes in T2DM patients and COPD patients (*p <0.05 and **p, ***p <0.01 vs. the control group).

According to the GSEA results, in T2DM, apart from Central memory CD8 T cells, Activated CD4 T cells, Type 1 T helper cells, Type 2 T helper cells, and Plasmacytoid dendritic cells, no other immunocytes showed a significant difference in their number between the two groups (**Figures 8A, C**). Notably, most COPD groups exhibited a higher immunocyte content than the control group. Specifically, 13/28 immunocytes (Activated CD8 T cells, Central memory CD8 T cells, Central memory CD4 T cells, Type 1 T helper cells, Activated B cells, Immature B cells, Myeloid-derived suppressor cells, Activated dendritic cells, Macrophages, Eosinophils, Mast cells, Monocytes, and Neutrophils) showed significant differences between the two groups (**Figures 8B, D**), with the difference in the levels of Central memory CD8 T cells and Type 1 T helper cells being more predominant. At the same time, the correlation between immunocytes and four core diagnostic genes was analyzed, revealing that PES1, CANX, and SUMF2 expressions correlated closely with most immunocytes in both the T2DM and COPD groups (**Figures 8E, F**).

### 3.7 Expression levels of shared DEGs in the single-cell transcriptome data

**Figure 9:**
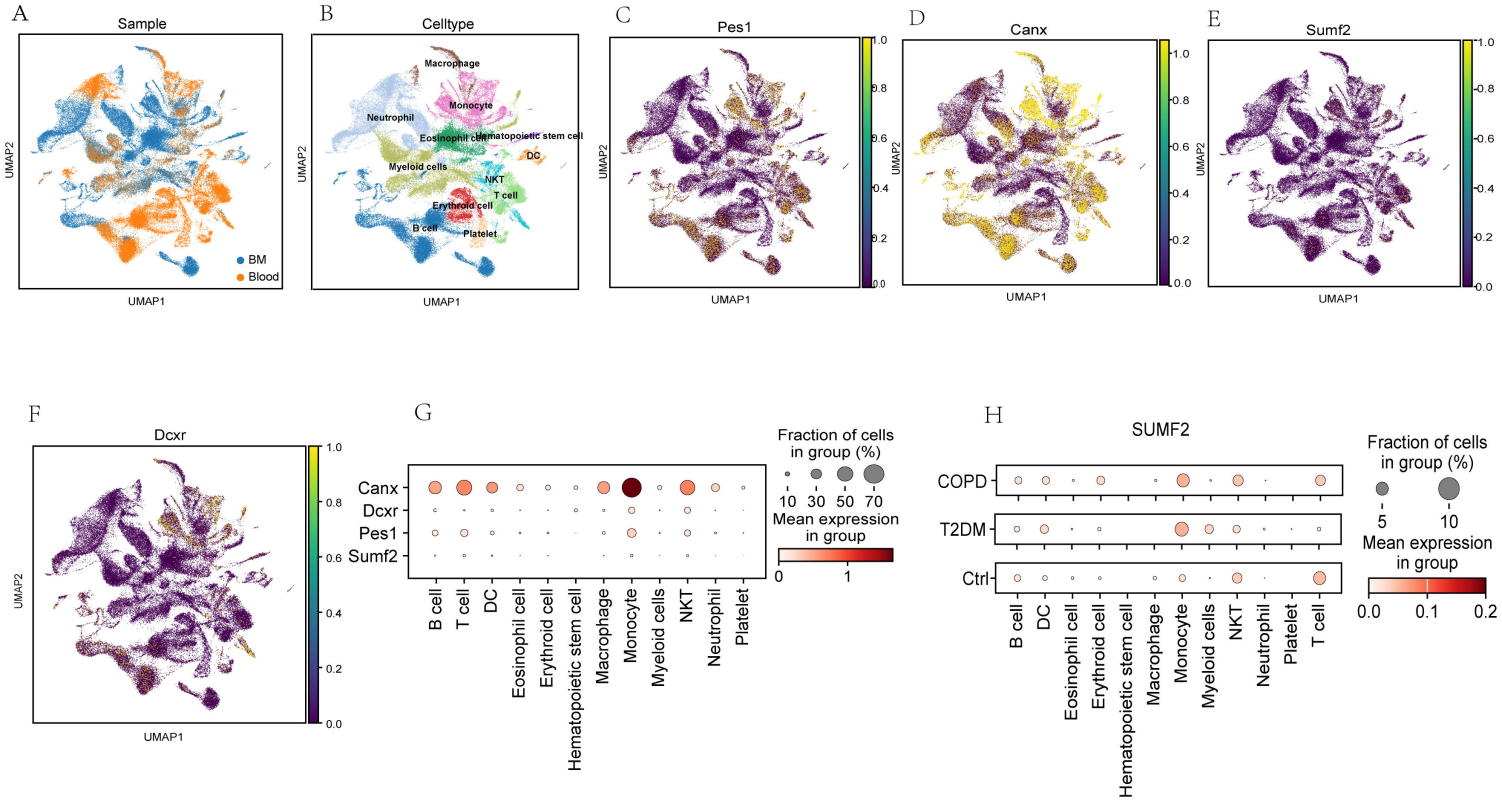
Analysis of the expression level of PES1, CANX, SUMF2, and DCXR in mouse single-cell transcriptome datasets (GSE216886, GSE212726 and GSE205078). **(A, B)** t-distribution random neighbor embedding (t-SNE) of 11 major cell types identified in three sets of single-cell transcriptome datasets. **(C–F)** t-SNE map of expressions of PES1, CANX, SUMF2, and DCXR. **(G)** Bubble chart showed PES1, CANX, SUMF2 and DCXR expressions in different cell types. The size of each point represent the percentage of expression; the average expression is indicated by color. **(H)** Single-cell transcriptome data showing the expression of CANX in 11 major immunocyte types in T2DM and COPD.

First, single-cell sequencing datasets for T2DM GSE216886 and COPD GSE205078, both of which are mouse samples, were downloaded from the NCBI GEO database. Subsequently, normalization, scaling, clustering, and highly variable gene screening were performed. A dimensionality reduction clustering 2D map was then generated based on the umap of these 2000 highly variable genes (**Figures 9A, B**). The cellular cluster expression maps of four DEGs shared between T2DM and COPD were also generated (**Figures 9C–F**). In addition, the Kruskal-Wallis test also validated the expression levels of DEGs common to T2DM and COPD in different immune cells, and although SUMF2 was expressed at a lower level in immune cells, by combining the RT-qPCR data of **Figure 7E** T2DM combined with COPD patients, it was found that compared with the healthy individuals, the expression level of SUMF2 alone was statistically different (**Figure 9G**). SUMF2 was downregulated in T cells in both COPD and T2DM groups (**Figure 9H**), which is consistent with all our previous human blood validation results.

## 4 Discussion

Given that the causal relationship between T2DM and COPD remains unclear, exploring their common features is crucial to unraveling potential associations. Exploring the pathophysiology, molecular mechanisms, clinical manifestations, and patient characteristics in T2DM and COPD to identify common diagnostic genes could yield novel insights, improve our understanding of the two diseases, and facilitate the development of cross-disease therapeutic strategies. In other words, identifying and recognizing the common features between T2DM and COPD could further elucidate the causal relationship between them.

Herein, the common disease pathways and diagnostic markers shared between T2DM and COPD were explored using bioinformatics analysis. The functional analysis of DEGs identified in different datasets revealed that the occurrence of both T2DM and COPD correlated closely with cellular senescence and immune-related pathways, especially those associated with Th1, Th2, and Th17 cell differentiation and Natural killer cell-mediated cytotoxicity. Moreover, the functional analysis identified disease-related modules and shared genes through gene cluster analysis, further confirming the important role of Th1, Th2, and Th17 cell differentiation in the pathogenesis of the two diseases. Based on these findings, we deduced that Th1, Th2, and Th17 cell differentiation could be involved in the pathogenesis of both T2DM and COPD. Additionally, there were 75 common genes between T2DM and COPD, which were subjected to LASSO regression, SVM-RFE, and RF analyses, revealing eight optimized core genes. Among them, PES1, CANX, SUMF2, and DCXR were identified as possible diagnostic markers for T2DM and COPD via PCR validation.

In a recent study, the T cell subpopulations Th1, Th2 and Th17, which are present in peripheral blood, were higher in T2DM patients than in healthy controls. Furthermore, each pro-inflammatory subpopulation exhibited a significant upregulation of T cell subpopulations, implying that cellular immunity and the polarization of T cell subpopulations toward the pro-inflammatory phenotype may contribute to the onset and progression of T2DM (10). According to recent research, COPD patients often exhibit immune function abnormalities, particularly characterized by a significant dysregulation in the proportion of the T lymphocyte subpopulation (11). Furthermore, T cells could accumulate in the respiratory tract and lung tissues, where they could secrete inflammatory cytokines and chemokines, potentially destroying lung tissues, thus leading to emphysema, a type of COPD (12). Based on these findings, it is plausible that T cells are a potential factor contributing to the pathogenesis of T2DM and COPD. Nonetheless, additional research is required to further elucidate the involvement of T cells in the pathogenesis of T2DM combined with COPD.

Herein, PES1, CANX, SUMF2, and DCXR were identified as potential diagnostic markers for T2DM and COPD. PES1 is a protein-coding gene initially identified in zebrafish embryos and plays a critical role in ribosome biogenesis, nucleolar generation, and cell proliferation. Previous studies have shown that PES1 regulates proteins associated with vascular permeability, thereby contributing to the amelioration of T2DM and other CVDs (13). Furthermore, PES1 was reported to ameliorate lipid dysregulation in T2DM (14, 15). Additionally, PES1 knockdown increased T-cell infiltration into subcutaneous tumors of Esophageal Squamous Cell Carcinoma (ESCC), promoting ESCC progression (6). According to research, DCXR belongs to the short-chain dehydrogenase/reductase superfamily and plays an essential role in glucose metabolism, particularly in the glucuronic acid/uric acid cycle pathway, a secondary route for glucose-6-phosphate oxidation (16). Studies have revealed that tobacco and active carbonyl compounds such as diacetyl (2, 3-butanedione) and 2, 3-pentanedione, which are present in many food products, could cause severe respiratory illnesses, and that carbonyl reductase in the lungs, especially DCXR, could detoxify most of these chemical substances (17). CANX is a companion protein present in the Endoplasmic Reticulum (ER), and its coding genes are adjacent to the MHC gene cluster. In a previous study, CANX was found to promote T cell activation and IFN-γ and TNF-α secretion by positively regulating MHC-1, thus enhancing the T cell killing effect on mouse tumor cells and immunocyte infiltration (18). SUMF2 is an important modifier that regulates Steroid Sulfatase (STS) activity. According to research, SUMF2 can inhibit the production of Th2 cytokines, thereby attenuating the inflammatory response, suggesting that SUMF2 may be associated with inflammation (19, 20). Our results showed that the expression levels of PES1, DCXR and CANX were decreased in T2DM and increased in COPD, while the expression level of SUMF2 was significantly decreased in both T2DM and COPD. This result suggests that these four key genes may play an important role in the immunoregulation of the two diseases, especially SUMF2, which may also play an important role in the immunoregulation of their comorbidities.

Whether PES1, CANX, SUMF2 and DCXR are diagnostic markers for T2DM and COPD was further demonstrated by analyzing single-cell sequencing data from blood and bone marrow, as well as RT-qPCR results from healthy individuals and patients with T2DM combined with COPD. The results showed that only SUMF2 had statistically different expression levels in comorbid patients compared to healthy individuals, while single-cell data showed that SUMF2 was significantly down-regulated in T cells in both the COPD and T2DM groups, which is consistent with all our validation results. Finally, combining the above results, we speculate that it may be due to the decrease of SUMF2 expression level, activation of T cells, which promotes the immune response and ultimately participates in the development of T2DM and COPD.

Repeated validation of SUMF2 further confirmed that there could be other key mechanisms underlying the correlation between SUMF2 and T2DM combined with COPD, highlighting a promising area for future research. Additionally, SUMF2 contributed to immunocyte infiltration, providing a potential target for more precise and personalized immunotherapy. Overall, our findings suggested that SUMF2 could be a potential diagnostic marker. In addition to providing a new strategy for the clinical diagnosis of T2DM combined with COPD, our findings also offer molecular targets for clinical diagnosis and drug development. However, considering the lack of *in vivo* validation in this study and the relatively limited sample size, especially in RT-qPCR analysis, *in vivo* validation and expansion of sample size in further studies are necessary to obtain more reliable conclusions. In addition, further functional studies are necessary to investigate the immunomodulatory role of SUMF2 in T cell subsets.

## 5 Conclusion

To the best of our knowledge, this is the first study to explore common pathways and genetic diagnostic markers for T2DM and COPD using bioinformatics analysis. Our findings suggested that T cell-related pathways may be associated with the pathogenesis of T2DM and COPD and that SUMF2 is a potential diagnostic marker for T2DM combined with COPD. Additionally, our immune infiltration correlation analysis revealed that the pathogenesis of T2DM and COPD may be closely related to an innate immune imbalance. Overall, this study presents a novel perspective for exploring the possible mechanisms underlying the pathogenesis of T2DM combined with COPD. Nonetheless, additional research involving relevant *in vitro* and *in vivo* experiments will be required to further explore the mechanisms of T cell-related pathways and SUMF2 expression changes in the two diseases.

## Data Availability

The datasets presented in this study can be found in online repositories. The names of the repository/repositories and accession number(s) can be found in the article/**Supplementary Material**.
