# Supplementary figures and images for: A comprehensive bioinformatics analysis of pathways and biomarkers shared between type 2 diabetes mellitus and chronic obstructive pulmonary disease
Source: Front Immunol. 2025 Jul 25;16:1536551. doi: 10.3389/fimmu.2025.1536551 (PMC12331674; doi:10.3389/fimmu.2025.1536551)

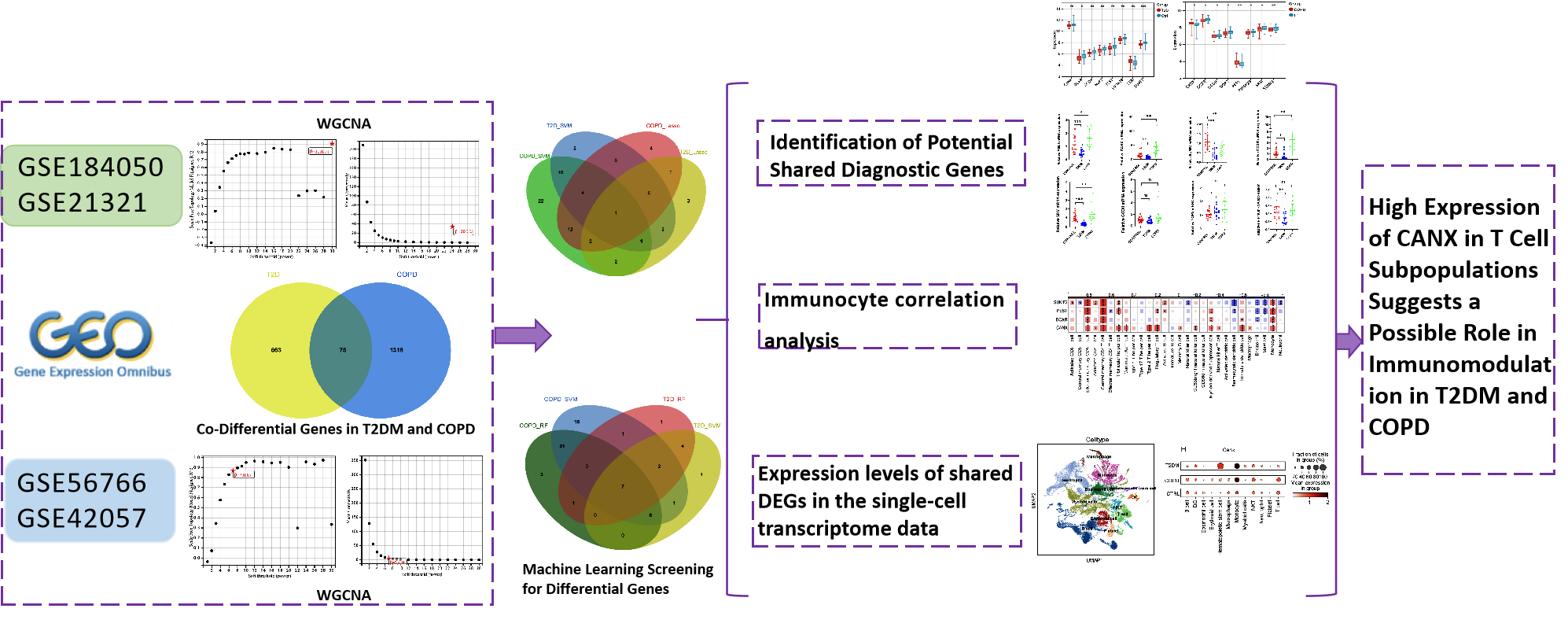


Supplemental Figure1: Visual Summary


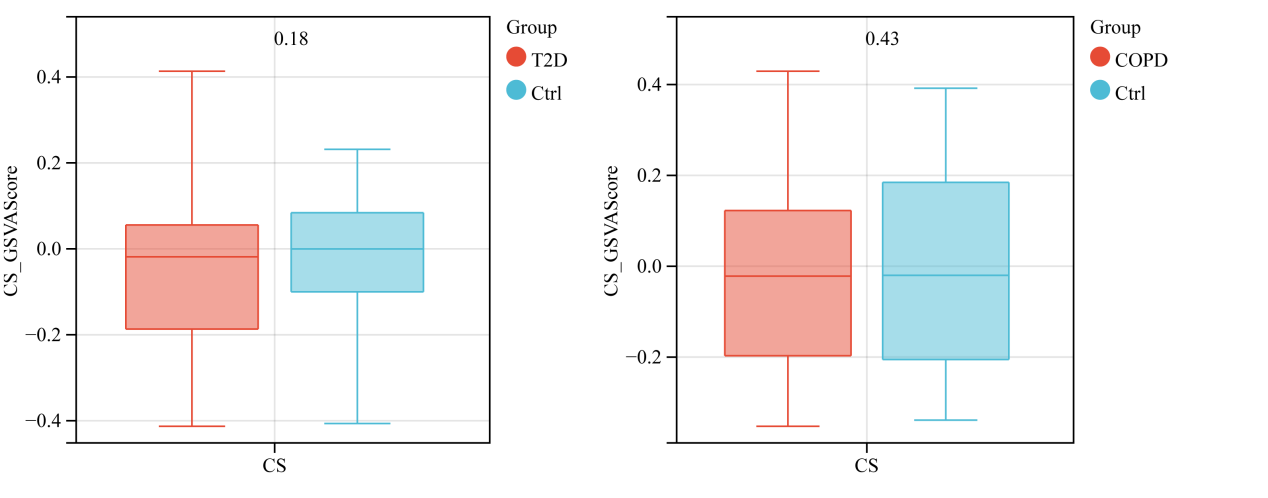


Supplemental Figure1:GSVA analysis for T2DM and COPD

Supplement: Supplementary file 1 [file Supplementaryfile1.docx]
